# Electrosprayed Alginate–Fmoc Amino Acid Microcapsules for Quercetin Loading and Release

**DOI:** 10.1021/acsomega.6c00004

**Published:** 2026-06-15

**Authors:** Hatice Asri, Serap Mert

**Affiliations:** † Department of Biomedical Engineering, Faculty of Technology, 52980 Kocaeli University, 41001 Kocaeli, Türkiye; ‡ Department of Chemistry, Faculty of Arts and Sciences, Kocaeli University, 41001 Kocaeli, Türkiye; § Department of Polymer Science and Technology, Kocaeli University, 41001 Kocaeli, Türkiye; ∥ Center for Stem Cell and Gene Therapies Research and Practice, Kocaeli University, 41001 Kocaeli, Türkiye

## Abstract

This study presents the development of hybrid microcapsules for the loading and release of quercetin, produced by incorporation of Fmoc-Tyrosine (Fmoc-Y) or Fmoc-Proline (Fmoc-Pro) into alginate, followed by Ca^2+^-ion-mediated cross-linking. The microcapsules produced using this method were characterized in terms of particle size, morphology, chemical structure (FTIR), release behavior, and swelling properties. An average diameter of microcapsules loaded with quercetin was measured at 187 ± 19 μm for Ca-Alginate, 154 ± 14 μm for Ca-Alginate/Fmoc-Y, and 131 ± 15 μm for Ca-Alginate/Fmoc-Pro. The inclusion of Fmoc-Pro or Fmoc-Y in the Ca-Alginate structure was shown by changes and shifts in the peak in FTIR. Rheological analyses revealed the flow behavior, viscoelastic properties, and shear-induced structural stability of alginate solutions used in microcapsule production. The incorporation of Fmoc-aa into alginate was intended to enhance the structural durability of the alginate matrix and thereby improve its drug release performance. Consistent with this expectation, examination of quercetin release at pH = 7.4 revealed that release from Ca-Alginate microcapsules was completed within 7 h, whereas release from the hybrid microcapsules extended to 24 h. Moreover, the zero-order model for Ca-Alginate microcapsules and the Korsmeyer–Peppas model for hybrid microcapsules were determined as the most appropriate mathematical models. The swelling behavior of the microcapsules was investigated at pH = 1.2 and 7.4. Although shrinkage occurred in the microcapsules at pH = 1.2, the spherical form was maintained in the first 7 h in hybrid and nonhybrid microcapsules. At pH = 7.4, it was observed that Ca-Alginate microcapsules were largely eroded approximately at the 6^th^ hour, while hybrid Ca-Alginate/Fmoc-Y and Ca-Alginate/Fmoc-Pro microcapsules maintained their spherical form up to 16 h. These findings are supported by SEM analyses showing that hybrid microcapsules exhibit a more compact and less porous surface morphology compared to pure Ca-Alginate microcapsules.

## Graphical abstract

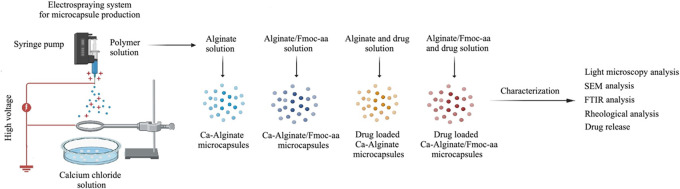

## 1 Introduction

Alginate is a biocompatible and naturally derived polymer that has attracted considerable attention in drug delivery due to its versatility in forming microcapsules,^1^ nanoparticles,^2^ nanofibers,^3^ and hydrogels.^4^ Its ability to form polymeric membranes makes alginate particularly suitable for controlled drug release systems, where encapsulation prevents burst release and enables sustained and efficient drug delivery.^2,5^ The choice of particle size is largely application-dependent; nanoparticle formulations are predominantly employed in cancer therapy,^6^ while microparticles are preferred for oral drug delivery,^7,8^ and tissue engineering applications.^9–13^ Consequently, alginate-based microcapsules are fabricated using different techniques such as ionic gelation,^14^ aerosolization,^5^ and electrospraying^9^ according to the targeted particle size and intended application.

Ca-Alginate and Ca-Alginate/chitosan microcapsules are widely used formulations for controlled drug release applications.^12,13,15,16^ The alginate microcapsules developed in these studies have commonly been evaluated in terms of their thermal properties using differential scanning calorimetry (DSC) and their drug release behavior in phosphate buffer at pH 7.4.^17,18^ The incorporation of chitosan into Ca-Alginate microcapsules has been shown to significantly enhance capsule durability.^19^ While drug-loaded alginate capsules without chitosan degrade within approximately 170 min at pH 7.4 (Simulated Colon Fluid, SCF),^14^ the addition of chitosan prolongs drug release up to 7 h, with capsule degradation occurring gradually through erosion after this period.^16^ In addition to improving mechanical stability, chitosan markedly enhances drug loading and encapsulation efficiency (EE). Alginate-only microcapsules reported in the literature exhibit relatively low drug loading (17%) and EE (37%), whereas chitosan-containing formulations achieve drug loading up to 55% and EE’s as high as 94%.^19^ However, chitosan is typically dissolved in acetic acid prior to its incorporation into alginate,^15^ which is undesirable for acid-sensitive drugs and biologically active molecules.^20^ Therefore, studies have explored the incorporation of fluorenylmethyloxycarbonyl-protected amino acids (Fmoc-aas) into alginate as an alternative to chitosan for enhancing structural integrity and controlling drug release.^21–23^

Fmoc-aas exhibit intrinsic self-assembly properties, which contribute to enhanced structural stability and improved control over drug release. The self-assembly behavior of Fmoc-aas arises from two key structural features: (i) the presence of both hydrophobic Fmoc groups and hydrophilic carboxyl (−COOH) moieties, which promote supramolecular organization, and (ii) the ability of deprotonated carboxylate groups (COO^–^), formed upon loss of a proton (H^+^), to interact and cross-link with divalent Ca^2+^ ions.^23^ Structural stabilization in Fmoc-aa-based systems is primarily achieved through noncovalent intermolecular interactions such as π–π stacking, hydrophobic interactions, and hydrogen bonds.^21–23^ These interactions facilitate the formation of a reinforced network within the alginate matrix. Therefore, alginate has been combined with various Fmoc-aas to form hybrid hydrogels and nanofibrous architectures.^21–23^ For example, the incorporation of Fmoc-tyrosine (Fmoc-Y) nanofibers into Ca-Alginate hydrogels enabled controlled release of Rhodamine B.^22^ Similarly, hybrid hydrogel beads composed of Ca-Alginate and Fmoc-diphenylalanine (Fmoc-FF) were developed for docetaxel delivery, exhibiting a slower release profile compared to beads containing only Fmoc-FF.^24^ Overall, these studies demonstrate that the addition of Fmoc-aa to alginate matrices enhances material durability and effectively retards drug release rates. Fmoc-aa-based systems are widely preferred in biomaterial applications due to their biocompatibility.^22,25^

Quercetin is widely recognized as a bioactive compound with well-documented antioxidant and anticancer properties.^26^ It belongs to the flavonoid family and is naturally found in various fruits, vegetables, and medicinal plants. Because of its strong free radical scavenging activity, quercetin plays a significant role in reducing oxidative stress, which is associated with numerous chronic diseases, including cancer. In addition to its antioxidant capacity, quercetin has roles as an anti-inflammatory, antibacterial, and anticancer agent. These characteristics make quercetin a promising therapeutic agent and an attractive model compound in pharmaceutical and biomedical research.^27^ In addition, quercetin has demonstrated significant therapeutic potential in the treatment of colon-related disorders such as ulcerative colitis and inflammatory bowel disease.^28–31^ Despite these promising biological activities related to the colon, the clinical efficacy of quercetin is limited by its low oral bioavailability and premature degradation in the gastrointestinal tract (GIT).^27^ Jing and co-workers have demonstrated that orally administered quercetin-loaded pectin/oligochitosan microcapsules enable colon-specific drug release and effectively alleviate colitis symptoms.^28^ Consequently, enhancing quercetin stability during gastric transit and enabling its targeted delivery to the intestine and colon are critical for improving therapeutic outcomes. Thus, in this study, we focused on developing more stable hybrid microcapsules by integrating Fmoc-Y or Fmoc-Pro into the alginate matrix to improve the release profile of quercetin. Therefore, quercetin-loaded hybrid microcapsules composed of Ca-Alginate/Fmoc-Y and Ca-alginate/Fmoc-Proline (Fmoc-Pro) (Figure 1) were developed, and the resulting microcapsules were comprehensively characterized in terms of particle size, morphology, FTIR analysis, EE, and swelling behavior, and their drug release profiles *in vitro* were evaluated in this study. The drug release mechanism of the developed microcapsules was analyzed by using zero-order, first-order, Higuchi, and Korsmeyer–Peppas models. The viscosity properties of alginate and Fmoc-aa solutions were characterized by rheometric analysis, and the effects of viscosity on microcapsule production were evaluated. The SEM images of free and quercetin-loaded Ca-Alginate, Ca-Alginate/Fmoc-Y, and Ca-Alginate/Fmoc-Pro microcapsules were evaluated in detail and analyzed comparatively. The swelling behavior of microcapsules in simulated gastric fluid (SGF) and SCF environments was investigated and was shown to exhibit different behaviors.

**Figure 1:**
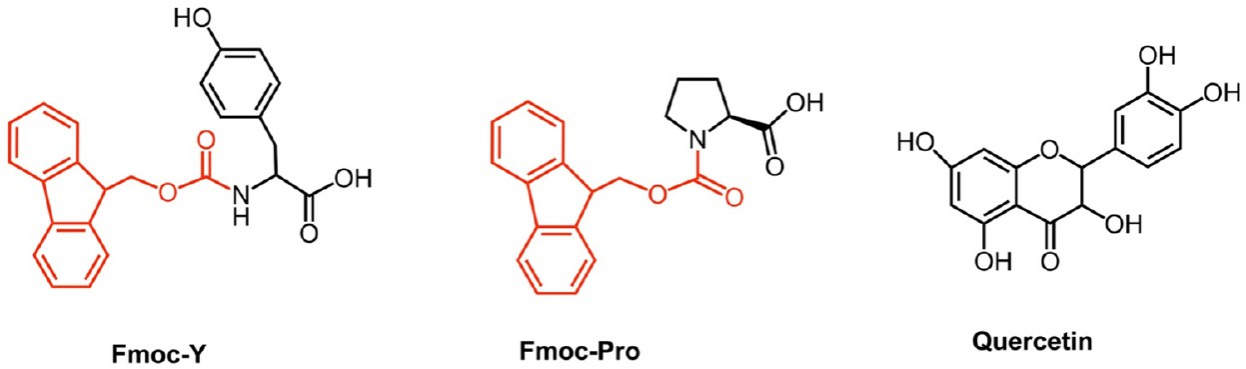
Structures of Fmoc-Y, Fmoc-Pro, and Quercetin.

## 2 Materials and Methods

### 2.1 Materials

Alginic acid sodium salt from brown algae (Sigma-Aldrich), CaCl_2_.2H_2_O (Chem Biotech), DMSO (dimethyl sulfoxide, ISOLAB), Fmoc-*L*-proline (Fmoc-Pro, Sigma-Aldrich), Fmoc-tyrosine (Fmoc-Y, BIOSYNTH Carbosynth), and quercetin (ChemCruz) were used to prepare alginate hybrid spheres. PBS solution (pH = 7.4) and HCl (pH = 1.2, 0.1 M) were prepared for the release medium. The shape and size of the microcapsules were obtained by optical microscopy (Olympus IX71), and the surface morphology by scanning electron microscopy (SEM) (Thermo Scientific, Quattro S SEM).

### 2.2 Methods

#### 2.2.1 Production of Ca-Alginate/Fmoc-aa Microcapsules by Electrospraying

Ca-Alginate, Ca-Alginate/Fmoc-Y, and Ca-Alginate/Fmoc-Pro hybrid microcapsules were produced by electrospraying using 2% w/v alginate (2 mL) and 3% w/v CaCl_2_ (150 mL) aqueous solutions prepared in deionized water. Fmoc-Y (5 mg) or Fmoc-Pro (2 mg) was dissolved in 75 μL of DMSO and a subsequently added to the alginate solution, and the resulting mixture was electrosprayed into 3% w/v CaCl_2_ solution according to the literature known protocol^9,32^ (Figure 2). During the production of all microcapsules, the alginate solution was dripped into the CaCl_2_ solution at a flow rate of 2 mL/h using a 27 G needle tip. Additionally, 18 kV voltage was applied between the needle tip and the ground.^33,34^ Microcapsules were produced by mixing at 150 rpm for 30 min after the completion of the dripping of the alginate solution into the CaCl_2_ solution.^34^ The obtained microcapsules were filtered through a 40 μm filter, collected, and lyophilized prior to storage at 4 °C until further analysis.

**Figure 2:**
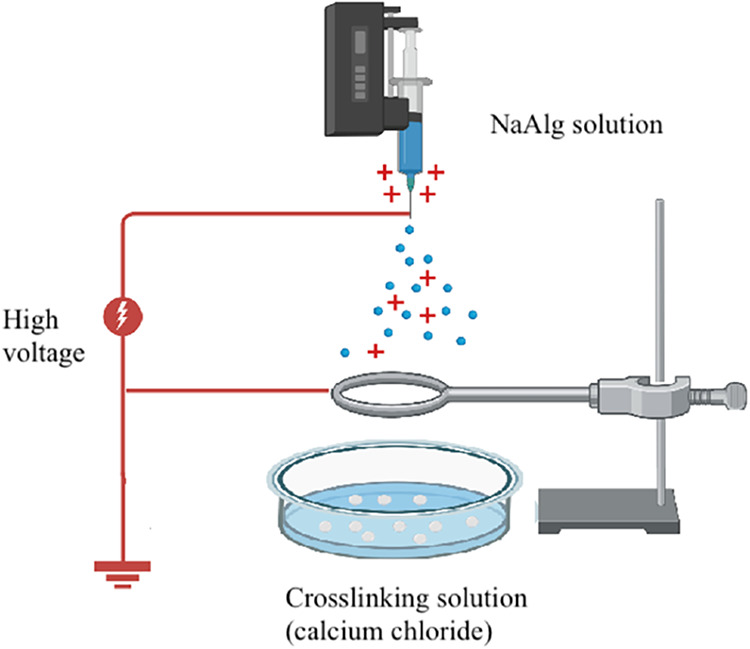
Electrospraying system (created with BioRender.com).

#### 2.2.2 Microcapsule Size and Morphology

The shape, size, and surface morphology of empty and quercetin-loaded Ca-Alginate, Ca-Alginate/Fmoc-aa microcapsules were characterized by optical microscopy (Olympus IX71) and scanning electron microscopy (SEM).^19,35^ The produced microcapsules were lyophilized prior to SEM analysis. For lyophilization, the filtered samples were first frozen at −20 °C overnight to ensure complete solidification of the aqueous phase.^9,36^ The prefrozen samples were subsequently transferred to a lyophilizer and dried under reduced pressure 10^–3^ mbar at −56 °C for approximately 48 h, allowing water removal via sublimation. This procedure was applied to preserve the morphology and structural integrity of the microcapsules and to minimize chemical or structural changes during drying.^37^ Lyophilization was selected as the drying method due to its suitability for alginate-based microcapsules.^34,38^ Additionally, microcapsules were imaged immediately after preparation in a wet (hydrated) state for SEM analysis. To minimize potential structural deformation, hydrated samples were imaged at approximately 0 °C under low-vacuum conditions (500–600 Pa) during SEM analysis (Figure S3). The resulting morphologies were compared with those of previously imaged lyophilized samples to evaluate possible differences in appearance.

#### 2.2.3 FTIR Analysis of Microcapsules

FTIR spectra were recorded using a Fourier transform infrared spectrometer equipped with an attenuated total reflectance (ATR) accessory. Measurements were performed in the range of 4000–400 cm^–1^ with a resolution of 4 cm^–1^, averaging 32 scans per sample. Freeze-dried microcapsules were placed directly on the ATR crystal to ensure proper contact during measurements.^39,40^

#### 2.2.4 Rheological Analyses

Rheological characterization of the samples was performed at a constant temperature of 25 °C using a rheometer (MCR). Before each measurement, the samples were rested for 5 min to allow them to reach thermal equilibrium and relieve stress generated during loading.^41^

##### 2.2.4.1 Flow Curve Test

The viscosity profiles of the solutions were determined by collecting 30 data points in the shear rate range of 0.01 to 1000 s^–1^. Power-law regression analysis was applied to describe the flow behavior of the obtained data; the consistency coefficient (K) and flow behavior index (n) values were calculated.^42^

##### 2.2.4.2 Thixotropy Test

Up–down flow scanning was performed to determine the time-dependent structural changes and recovery capacities of the samples. The hysteresis area resulting from this cycle of increasing and decreasing the shear rate was calculated.^43^

##### 2.2.4.3 Amplitude Sweep

Amplitude sweep was performed at a constant frequency of 1 Hz in the strain range of 0.01 to 100% to determine the linear viscoelastic (LVE) region of the samples. This test examined the strain-dependent changes in the storage modulus (*G*′) and loss modulus (*G*″) structural stability limits.^43^

#### 2.2.5 Determination of Encapsulation Efficiency and Drug Release from Microcapsules

Quercetin-loaded Ca-Alginate microcapsules were incubated in 1 mL PBS solution at pH = 7.4, 100 rpm, 37 °C. At 30, 60, 90 min, 2, 3, 4, 5, 6, 7, and 24 h intervals, 1 mL of PBS was withdrawn from the release medium of quercetin-loaded Ca-Alginate microcapsules, and fresh PBS was added and the process was repeated for each time interval. For the UV analysis of quercetin, standard quercetin solutions were prepared in the concentration range of 0.15–40 μg/mL using the same release medium employed in the *in vitro* studies. Due to the low solubility of quercetin in PBS, a PBS:DMSO (v/v, 4/1) mixture was used as the solvent. The UV measurements were carried out at 385 nm, and the calibration curve was constructed by plotting absorbance versus concentration (Figure S2).^18,44^ The collected release samples were analyzed by UV spectrophotometer at 385 nm wavelength.^18,45^ The EE values of quercetin in microcapsules were calculated from the total drug amount released. EE was calculated according to the following equation: EE (%) = (loaded drug amount/initial drug amount) × 100.^46,47^

#### 2.2.6 Swelling Behavior of Microcapsules

To examine the swelling behavior of the microcapsules, microcapsules were incubated at 37 °C, 100 rpm, pH = 1.2 (in HCl solution),^14,19^ and pH = 7.4 (in PBS).^1,18,19^ Samples taken at certain time intervals (30 min, 1, 2, 3, 4, 5, 6, 7, 16, and 24 h) were examined under a light microscope. Spherical erosions and degradation were analyzed from microscope images.

## 3 Results and Discussion

### 3.1 Investigation of the Size and Morphology of Microcapsules Using Light Microscopy and Scanning Electron Microscopy (SEM)

Ca-Alginate, Ca-Alginate/Fmoc-Y, and Ca-Alginate/Fmoc-Pro hybrid microcapsules were produced by the electrospraying method. In order to determine the optimum conditions, the production of Ca-Alginate microcapsules was carried out by varying the physical parameters (Table 1). The alginate solution was dripped into the CaCl_2_ solution using a 27 G needle tip during the production of all microcapsules.^32^ A voltage of 18 kV is applied between the needle tip and ground.^34^ In the literature, it has been reported that the average size of Ca-Alginate microcapsules obtained using an alginate concentration of 2% w/v (2 mL) and a CaCl_2_ concentration of 3% w/v is approximately 100–400 μm.^9,32^ Therefore, alginate concentration was determined as 2% w/v (2 mL) and CaCl_2_ concentration as 3% w/v in this study. The experiments were carried out without and with the use of a ring electrode in the production of Ca-Alginate and Ca-Alginate/Fmoc-Y and Ca-Alginate/Fmoc-Pro microcapsules. In the microcapsules produced without the use of a ring electrode, the distance between the needle tip and CaCl_2_ solution was set to 16 cm as stated in the literature^9^ (Capsule 1, Table 1, Figure 3). In the production of microcapsules using a ring electrode, the distance between the needle tip and the ring electrode was set to 5 cm.^48^ The ring electrode increased the dripping frequency, decreasing the droplet size and resulting in spheres that were closer in size (Capsule 2, Table 1, Figure 3). Calcium alginate microcapsules were produced by dripping alginate solution at different flow rates (2 mL/hour and 5 mL/hour) from a syringe pump, and it was observed that the diameter of the capsules obtained at a flow rate of 2 mL/hour decreased to 186 ± 14 μm (Capsule 3, Table 1, Figure 3), and the flow rate was determined to be 2 mL/hour.^33,34^ As reported in the literature, decreasing the flow rate decreased the diameter of the microcapsules.^48^ Ca-Alginate microcapsules were produced with an average diameter of 186 ± 14 μm under these conditions.

**Figure 3:**
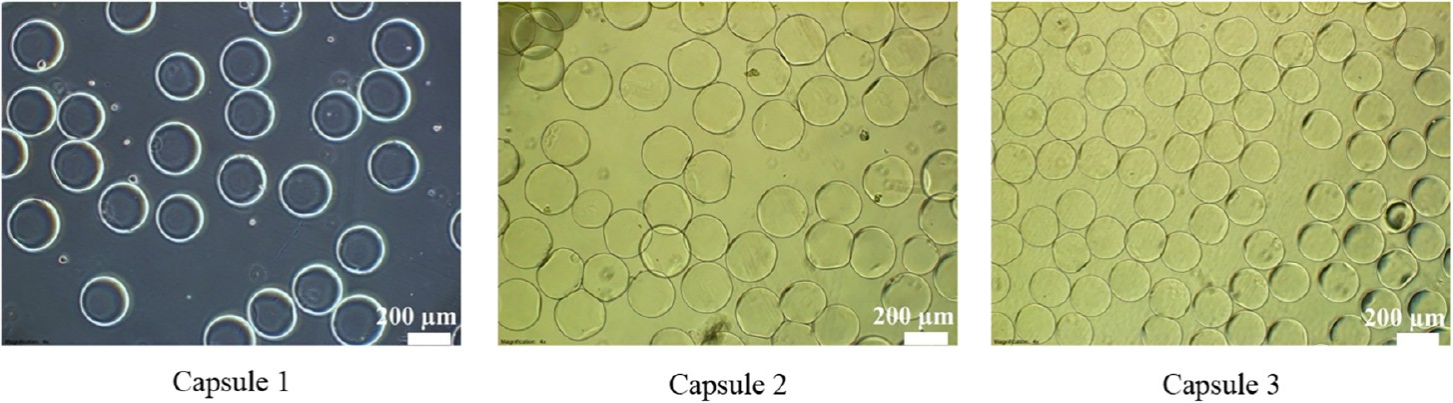
Microcapsules produced by the electrospraying method without addition of DMSO (Capsules 1–3, Table 1).

**Table 1:** Electrospraying Process Parameters for the Fabrication of Ca-Alginate, Ca-Alginate/Fmoc-Y, and Ca-Alginate/Fmoc-Pro Microcapsules^a^,^b^

**Capsule no**	**Flow rate** (mL/h)	**Voltage (kV)**	**Needle-ground distance (cm)**	**DMSO amount (μL)**	**Fmoc-aa type/amount (mg)**	**Drug amount (mg)**	**Average size distribution** ^c^ **[μm]**
Capsule 1	5 mL/h	18	16	-	-	-	238 ± 13 μm
Capsule 2	5 mL/h	18	5 (ring electrode)	-	-	-	212 ± 25 μm
Capsule 3	2 mL/h	18	5 (ring electrode)	-	-	-	186 ± 14 μm
Capsule 4	2 mL/h	18	5 (ring electrode)	75	-	-	168 ± 13 μm
Capsule 5	2 mL/h	18	5 (ring electrode)	75	-	1 mg	187 ± 19 μm
Capsule 6	2 mL/h	18	5 (ring electrode)	75	Fmoc-Y/5 mg	-	159 ± 16 μm
Capsule 7	2 mL/h	18	5 (ring electrode)	75	Fmoc-Y/5 mg	1 mg	154 ± 14 μm
Capsule 8	2 mL/h	18	5 (ring electrode)	75	Fmoc-Pro/2 mg	-	114 ± 14 μm
Capsule 9	2 mL/h	18	5 (ring electrode)	75	Fmoc-Pro/2 mg	1 mg	131 ± 15 μm

a Alginate 2% (w/v).

b CaCl_2_ 3% (w/v).

c The average of 20 measured spheres.

In the preparation of Ca-Alginate/Fmoc-Y microcapsules, Fmoc-Y (5 mg) was dissolved in DMSO (75 μL) and added to a 2% (w/v, 2 mL) alginate solution under stirring at 1000 rpm. After 30 min of stirring to obtain a homogeneous mixture, the solution was electrosprayed into 3% (w/v, 150 mL) CaCl_2_ solution, yielding Ca-Alginate/Fmoc-Y microcapsules with an average diameter of 159 ± 16 μm (Capsule 6, Table 1). Notably, Capsule 6 exhibited a smaller diameter than Capsule 3 (Table 1), suggesting that the presence of DMSO may influence microcapsule size. To clarify the effect of DMSO, alginate-only microcapsules were prepared in the presence of DMSO without Fmoc-aa (Capsule 4, Table 1). When compared to Capsule 3, which was prepared without DMSO, Capsule 4 displayed an approximately 20 μm reduction in average diameter. These findings consistently indicate that the incorporation of DMSO contributes to the formation of smaller microcapsules, likely through its influence on solution properties during the electrospraying process. According to the literature, the diameter of electrosprayed droplets is strongly dependent on solution concentration and viscosity, with lower viscosities generally leading to the formation of smaller microcapsules.^49^ The incorporation of DMSO may have reduced the overall viscosity of the solution, thereby facilitating droplet breakup under the applied electric field and resulting in decreased microcapsule diameters. Moreover, DMSO’s low surface tension promotes the formation of smaller microcapsules during the electrospray process. Furthermore, the presence of DMSO may have influenced intermolecular interactions, which could also contribute to the reduction in microcapsule size.

The size of the microcapsules plays an important role in the application area; as the size of the capsules decreases, the resistance to shear and compressive pressures increases and durability increases. Microcapsules produced below 200 μm were found to be easier to pass through the GIT. For this purpose, microcapsules below 200 μm were produced by the electrospraying method in drug release studies.^32,33^ In the preparation of drug (quercetin)-loaded Ca-Alginate microcapsules, quercetin (1 mg) was dissolved in DMSO (75 μL) and added to the alginate solution, stirred to get a homogeneous mixture. Subsequently, the same protocol was followed for the preparation of microcapsules, yielding microcapsules with an average diameter of 187 ± 19 μm (Capsule 5, Table 1, Figure 4). Quercetin Ca-Alginate/Fmoc-Y microcapsules were obtained with an average diameter of 154 ± 14 μm under the same condition (Capsule 7, Table 1, Figure 4).

**Figure 4:**
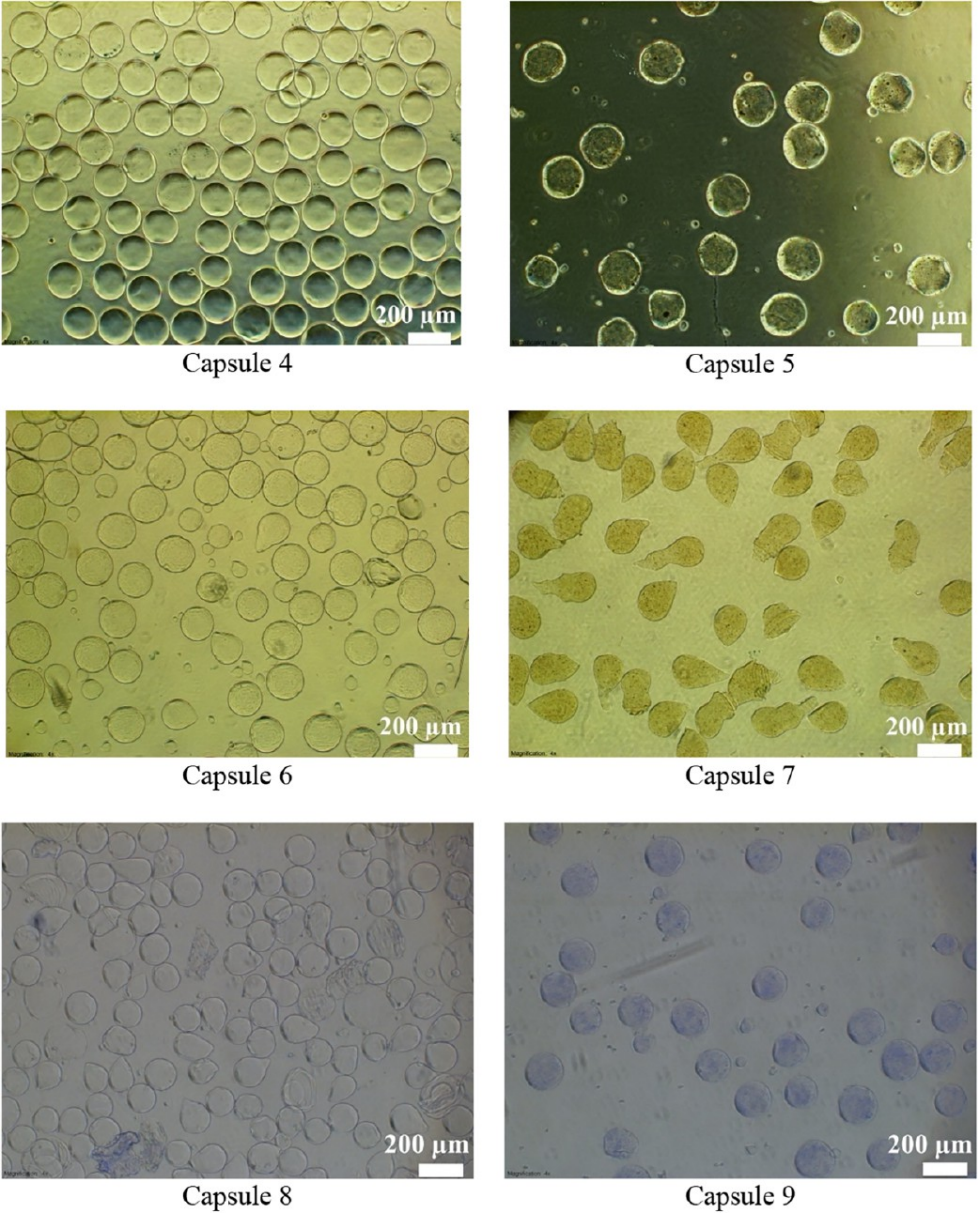
Light microscopy images of empty and quercetin-loaded Ca-Alginate, Ca-Alginate/Fmoc-Y, and Ca-Alginate/Fmoc-Pro microcapsules produced by the electrospraying method (Capsules 4–9, Table 1).

The preparation of Ca-Alginate/Fmoc-Pro microcapsules followed the same protocol as that used for Ca-Alginate/Fmoc-Y microcapsules. However, when the Fmoc-Pro content was increased to 5 mg, the solution exhibited greater resistance to the 18 kV electric field during electrospraying, resulting in the formation of oval-shaped microcapsules. It is well established that the geometry of electrosprayed droplets and the resulting microstructures is strongly influenced by solution concentration and viscosity.^49,50^ The observed oval-shaped microcapsules can thus be attributed to an increase in viscosity upon the addition of Fmoc-Pro, likely caused by enhanced intermolecular interactions between Fmoc-Pro and alginate. Fmoc-Pro contains a hydrophobic aromatic ring capable of π–π interactions and a hydrophilic carboxyl (−COOH) group, enabling strong hydrogen bonding and self-assembly behavior.^24,51^ These combined interactions modify the rheological properties of the solution, increasing its resistance to jet breakup under the applied electric field and leading to oval-shaped microcapsules. Reducing the Fmoc-Pro concentration eliminated this resistance, and 2 mg was selected as the optimal amount (Capsules 8 and 9, Table 1). The average diameter of Ca-Alginate/Fmoc-Pro microcapsules (Capsules 8 and 9, Table 1) was smaller than that of Ca-Alginate/Fmoc-Y microcapsules (Capsules 6 and 7, Table 1) (Figure 4).

SEM analysis of empty and quercetin-loaded Ca-Alginate (Capsules 4 and 5, Table 1), Ca-Alginate/Fmoc-Y (Capsules 6 and 7, Table 1), and Ca-Alginate/Fmoc-Pro (Capsules 8 and 9, Table 1) was performed at different magnifications after freeze-drying (Figures 5 and S1). Ca-Alginate microcapsules (Capsules 4 and 5, Table 1) were compared with Ca-Alginate/Fmoc-Y (Capsules 6 and 7, Table 1) and Ca-Alginate/Fmoc-Pro microcapsules (Capsules 8 and 9, Table 1), and differences were observed, particularly in surface structure (Figures 5 and S1). SEM images reveal layered structures, particularly in Ca-Alginate/Fmoc-aa microcapsules (Capsules 6–9, Figures 5 and S1). These layered structures are more clearly visible in empty and drug-loaded Ca-Alginate/Fmoc-Y microcapsules (Capsules 6 and 7, Figure S1). To evaluate the morphology of the microcapsules in greater detail, SEM analysis was performed on low-vacuum-dried and wet (hydrated) microcapsules under wet conditions in addition to lyophilized samples (Figure 5). When the SEM images of the lyophilized and wet microcapsules were compared with the wet SEM images, it was observed that all lyophilized microcapsules shrank because of the removal of water. Following freeze-drying and the low-vacuum drying process applied for SEM measurements, drug-loaded and drug-free Ca-Alginate microcapsules (Capsules 4, 5) were found to exhibit a more significant reduction in diameter compared to the Ca-Alginate/Fmoc-aa (Capsules 6–9) microcapsules. This suggests that microcapsules containing Fmoc-aa in their structure have a lower water content and, consequently, undergo a smaller degree of shrinkage. Moreover, the images obtained from wet SEM analysis showed a significant decrease in porosity in Ca-Alginate microcapsules (Capsules 4, 5) compared to Ca-Alginate/Fmoc-aa microcapsules (Capsules 6–9). Ca-Alginate/Fmoc-Y microcapsules (Capsules 6, 7) with a porous structure exhibited a narrower and more compact morphology compared to Ca-Alginate microcapsules. These morphological observations in quercetin-loaded Ca-Alginate/Fmoc-aa microcapsules support a slower and longer-lasting drug release.^19^

**Figure 5:**
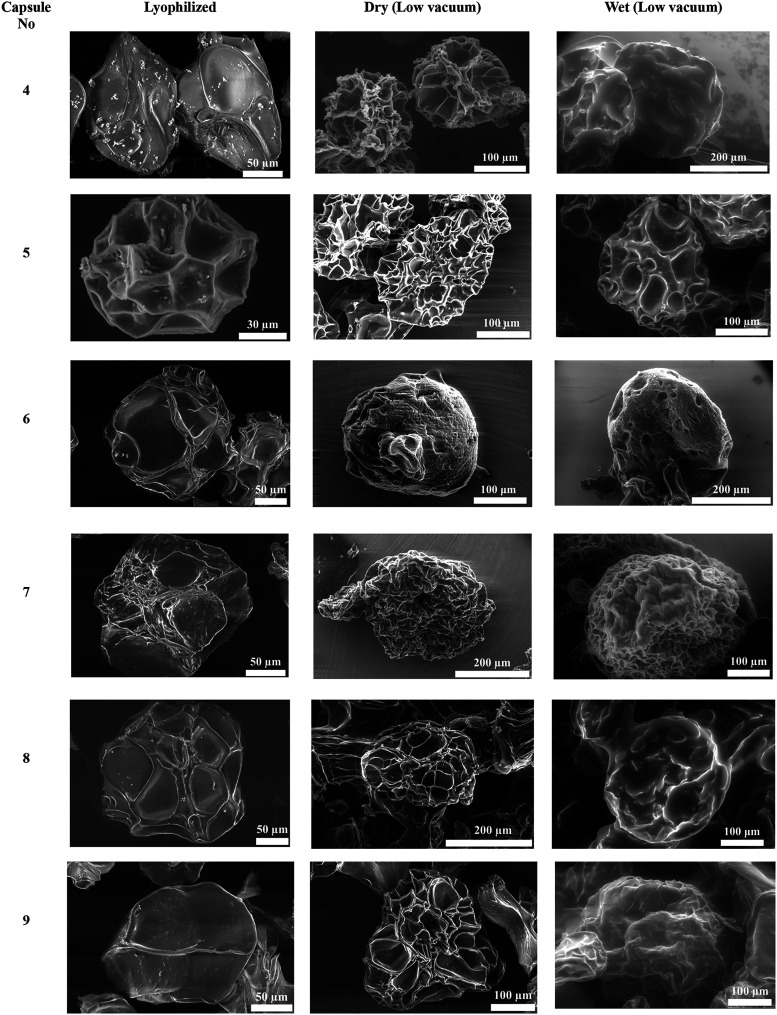
SEM analysis of microcapsules at different magnifications and conditions.

### 3.2 FTIR Analysis

In the FTIR spectrum of pure quercetin, the stretching of −OH groups is detected at 3398 cm^–1^ and 3279 cm^–1^. The peak at 1378 cm^–1^ indicates phenolic −OH bending. Stretching of C=O aryl ketonic is observed at 1663 cm^–1^. C=C aromatic ring stretching bands are detected at 1605, 1561, and 1519 cm^–1^. The in-plane bending band of C–H in the aromatic hydrocarbon is observed at 1313 cm^–1^, and the out-of-plane bending bands are detected at 937, 817, 670, and 598 cm^–1^. The bands at 1259, 1198, and 1163 cm^–1^ can be attributed to C–O stretching in the aryl ether ring, C–O stretching in phenol, and C–CO–C stretching and bending in ketone (Figure 6).^52^ In the FTIR analysis of alginate, the sharp peak at 1592 cm^–1^ indicates symmetric stretching of the carboxyl peaks (COO^–^), the sharp bands at 1403 cm^–1^ indicate asymmetric stretching of the carboxyl peaks (COO^–^), and the broad band at 3205 cm^–1^ indicates stretching of the (OH) group. The band observed at 1022 cm^–1^ corresponds to C–O stretching, and 1296 cm^–1^ is assigned to (OH) bending (Figure 6).^2,53^ According to the results of FTIR analysis of Fmoc-Pro, amide I bond due to C=O stretching at 1646 cm^–1^ and amide II band at 1523 cm^–1^ were determined. C=C, C–C, and C–H aromatic rings were determined at 962, 884, and 732 cm^–1^. In the FTIR analysis of Fmoc-Y, amide I bond due to C=O stretching at 1652 cm^–1^ and amide II band at 1535 cm^–1^ are detected. C=C, C–C, and C–H aromatic rings are observed at 979, 899, and 736 cm^–1^ (Figure 6).^54^

**Figure 6:**
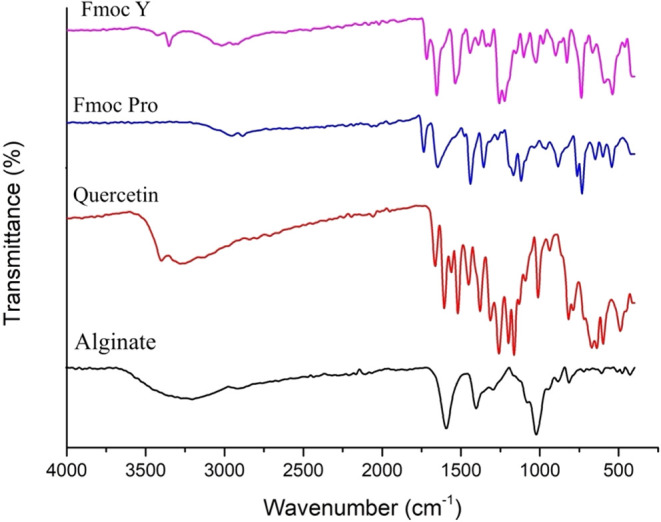
FTIR spectra of Fmoc-Y, Fmoc-Pro, Quercetin, and Alginate.

FTIR spectroscopy was employed to analyze the characteristic functional groups of Fmoc-Y and Fmoc-Pro within the alginate-based microcapsules (Capsules 6–9, Table 1). In the FTIR analysis, the carboxyl peak at 1592 cm^–1^ in alginate shifted to 1589 cm^–1^ in the empty Ca-Alginate microcapsules (Capsule 4, Table 1). This is attributed to the strong electrostatic interaction between Ca^2+^ ions and the carboxylic groups of alginate (Figure 7).^55,56^ The wavenumber at 1589 cm^–1^ in empty Ca-Alginate microcapsules (Capsule 4, Table 1) shifted to 1618 and 1596 cm^–1^ in empty Ca-Alginate/Fmoc-Pro microcapsules (Capsule 8, Table 1) and empty Ca-Alginate/Fmoc-Y microcapsules (Capsule 6, Table 1) due to the amide I bond due to C=O stretching.^54^ Additionally, the COO^–^ asymmetric peak (1403 cm^–1^) in alginate showed a shift toward higher wavenumbers (1411 cm^–1^) in empty Ca-Alginate microcapsules (Capsule 4, Table 1) (Figure 7).^57^ The peak at 1411 cm^–1^ in empty Ca-Alginate microcapsules (Capsule 4, Table 1) shifted to 1418 cm^–1^ in empty Ca-Alginate/Fmoc-Pro microcapsules (Capsule 8, Table 1) and 1422 cm^–1^ in Ca-Alginate/Fmoc-Y microcapsules (Capsule 6, Table 1).^54^ The peak at 1618 cm^–1^ in empty Ca-Alginate/Fmoc-Pro microcapsules (Capsule 8, Table 1) shifted to 1623 cm^–1^ (Figure 7). The peak at 1596 cm^–1^ in empty Ca-Alginate/Fmoc-Y microcapsules (Capsule 6, Table 1) shifted to 1589 cm^–1^ in quercetin-loaded Ca-Alginate/Fmoc-Pro microcapsules (Capsule 7, Table 1) due to C=O aryl ketonic absorption in pure quercetin (Figure 7).^52^ The results obtained support the interaction of Fmoc-aa in Ca-Alginate/Fmoc-Y and Ca-Alginate/Fmoc-Pro hybrid microcapsules and the loading of quercetin in Ca-Alginate/Fmoc-Y and Ca-Alginate/Fmoc-Pro hybrid microcapsules.

**Figure 7:**
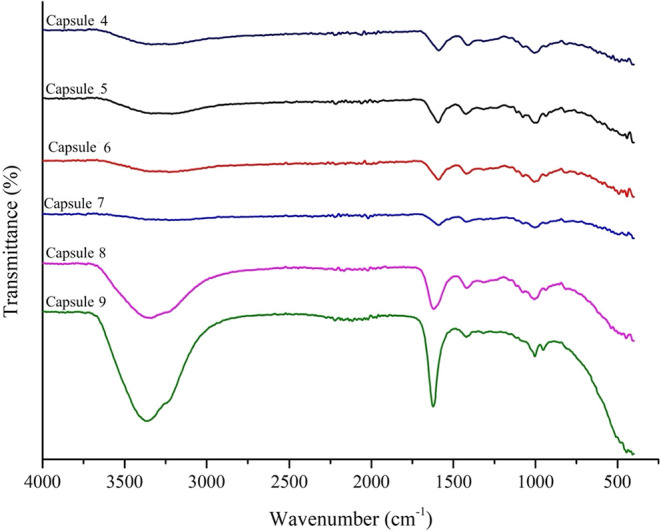
FTIR spectra of Ca-Alginate (Capsule 4), Quercetin-loaded Ca-Alginate (Capsule 5), Ca-Alginate/Fmoc-Y (Capsule 6), Quercetin-loaded Ca-Alginate/Fmoc-Y (Capsule 7), Ca-Alginate/Fmoc-Pro (Capsule 8), Quercetin-loaded Ca-Alginate/Fmoc-Pro (Capsule 9) microcapsules (sample details are provided in Table 1).

### 3.3 Rheological Analyses Results

Within the scope of the rheological analyses performed, flow curve, amplitude scanning, and thixotropy tests were evaluated together to reveal the flow behavior, viscoelastic properties, and time-dependent structural changes of the samples. Flow parameters (*K* and *n*) determined by the power-law model described the shear rate-dependent behavior of the systems, while *G*′–*G*″ analyses provided information about structural stability (Figure 8). In addition, hysteresis field results allowed for a comparative evaluation of the structural degradation and remodeling capacities of the samples under shear.

**Figure 8:**
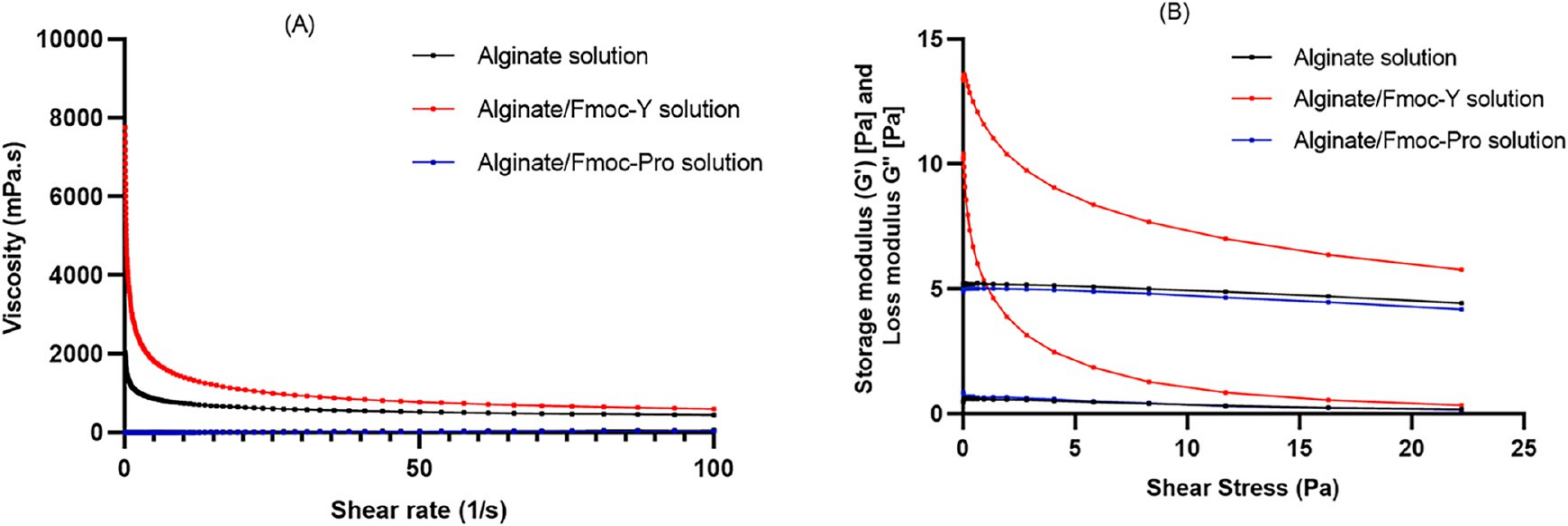
Rheological analyses of Alginate, Alginate/Fmoc-Y, and Alginate/Fmoc-Pro solutions: (A) flow curve test and (B) amplitude sweep results.

#### 3.3.1 Flow Curve Test

According to power-law regression analysis, both Alginate (*n* = 0.78, *K* = 1.23 × 10^3^) and Alginate/Fmoc-Y solutions (*n* = 0.63, *K* = 3.31 × 10^3^) exhibit shear-thinning (pseudoplastic) behavior (*n* < 1). However, Alginate/Fmoc-Y has a lower *n* value and a higher *K* value, resulting in a more viscous structure at lower shear speeds and a faster thinning of the material as shear increases. Alginate solution, on the other hand, exhibits a more fluid structure and weaker shear-thinning behavior. This situation supports the production of Alginate/Fmoc-Y solution in smaller sizes compared to Ca-Alginate solution using the electrospraying method. The *n* > 1 value for Ca-Alginate/Fmoc-Pro (*n* = 1.78) indicates shear-thickening behavior, showing that the flow resistance of the system increases as the shear rate increases (Figure 8A).^41,42^

#### 3.3.2 Amplitude Sweep

In the Alginate and Alginate/Fmoc-Pro (2) solutions, the *G*′ values are around 5 Pa and show only a slight decrease with increasing stress/strain. This indicates that both samples exhibit a low-modulus but more stable viscoelastic structure. In addition, the LVE region appears wider in these two samples; that is, the structure can remain intact for a longer time against the applied deformation. In the Alginate/Fmoc-Y solutions, the initial *G*′ value is significantly higher. This suggests that the addition of Fmoc-Y creates a stronger and more elastic network structure in the alginate system. However, in this sample, the *G*′ and *G*″ values decrease more rapidly with increasing stress/strain. Therefore, although the initial mechanical strength of Alginate/Fmoc-Y is higher, the LVE region is narrower and the structure is more susceptible to deformation (Figure 8B).^43^

#### 3.3.3 Thixotropy Test

The Alginate/Fmoc-Y solution showed the highest hysteresis field (*A* = 225.15 Pa/s), indicating a more pronounced thixotropic behavior. Pure alginate solution exhibited a moderate hysteresis field (*A* = −46.855 Pa/s), while Alginate/Fmoc-Pro had a lower hysteresis field (*A* = −26.686 Pa/s) and showed a more stable structure. The ranking based on absolute values is Alginate/Fmoc-Y > Alginate > Alginate/Fmoc-Pro solutions. This result shows that Fmoc-Y causes the system to exhibit a more pronounced tendency toward structural distortion and rearrangement under shear forces, while Fmoc-Pro creates a more stable network structure by increasing the post-shear recovery capability.^43^

### 3.4 Determination of Encapsulation Efficiency and Drug Release from Microcapsules

The EE was 90 ± 10% in Ca-Alginate microcapsules (Capsule 5, Table 2), 76 ± 3% in Ca-Alginate/Fmoc-Y microcapsules (Capsule 7, Table 2), and 78 ± 4% in Ca-Alginate/Fmoc-Pro microcapsules (Capsule 9, Table 2). The EE of quercetin was higher in Ca-Alginate microcapsules (Capsule 5, Table 2) than in Ca-Alginate/Fmoc-Pro microcapsules (Capsules 7 and 9, Table 2). In this study, the incorporation of Fmoc-aa likely altered key solution properties, particularly viscosity, which may have contributed to the observed decrease in EE. Oval-shaped Alginate/Fmoc-aa microcapsules were formed (Capsule 7, Figure 4) during electrospraying, and the literature indicates that this geometry is associated with solution viscosity.^49,50^ Changes in viscosity can affect droplet breakup and uniform jet formation, thereby influencing microcapsule geometry and potentially reducing EE. When preparing micro- or nanoparticles using the electrospray method, the viscosity of the matrix solution is a key parameter that significantly influences EE. During electrospray production, factors such as low viscosity, high applied voltage, and the formation of small droplets can lead to diffusion loss of the active ingredient before cross-linking, resulting in relatively lower EE. This mechanism can limit active substance retention by affecting jet stability and droplet surface area during the electrospray process.^58^ In particular, solution viscosity, and applied voltage not only influence particle morphology but also govern the encapsulation of the active ingredient within the matrix. Low-viscosity solutions can promote the dispersion of the active ingredient into the surrounding medium without sufficient incorporation into the matrix, due to the formation of small droplets with a high surface-to-volume ratio; this may contribute to lower EE values.^59^ Accordingly, the relatively low EE observed in Ca-Alginate/Fmoc-aa microcapsules produced by electrospraying can be attributed to method-specific droplet formation and differences in solution viscosity. In this case, EE could be influenced by the solvent volumes and ratios. The amount of DMSO cannot be reduced below the level required to maintain the solubility of Fmoc-aa. However, increasing DMSO or water content while simultaneously decreasing the polymer concentration to modify solution viscosity. Under such conditions, variations in solvent composition and viscosity properties can significantly influence droplet formation, internal microcapsule structure, and intermolecular interactions during electrospraying. Reduced viscosity often leads to the formation of less compact and more porous microcapsules, which can accelerate diffusion of the encapsulated compound. It was concluded that further reducing the viscosity would be inappropriate, as low viscosity could result in both a decrease in EE and an accelerated drug release. Additionally, the presence of Fmoc-aa in the medium may have facilitated interactions between Fmoc-aa molecules and alginate, while reducing the molecular interaction between the drug and alginate, which could have contributed to the decrease in EE. High quercetin EE may be achieved by reducing the Fmoc-aa/Alginate ratio even further and precisely adjusting the viscosity of the solution. However, it is anticipated that the reduction of the Fmoc-aa concentration within the hybrid microspheres could compromise the self-assembly behavior of Fmoc-aa, which plays a key role in stabilizing the internal structure.^22,24^ A loss of this supramolecular organization may, in turn, negatively affect the sustained release profile of quercetin. Therefore, an optimal balance between improving EE and preserving the self-assembly-driven slow-release characteristics must be carefully considered in future formulations.

**Table 2:** Ca-Alginate, Ca-Alginate/Fmoc-Y, and Ca-Alginate/Fmoc-Pro Hybrid Microcapsules Produced by Electrospraying^a^,^b^,^c^

**Capsule no**	**Fmoc-aa type and amount (mg)**	**Drug amount (mg)**	**Average size distribution [μm]**	**Average obtained microcapsule amount (mg)**	**Loading amount of quercetin [μg]**	**EE [%]**
Capsule 4	-	-	168 ± 13 μm	24 ± 8	-	-
Capsule 5	-	1 mg	187 ± 19 μm	30 ± 5	900 ± 100	%90 ± 10
Capsule 6	Fmoc-Y/5 mg	-	159 ± 16 μm	30 ± 11	-	-
Capsule 7	Fmoc-Y/5 mg	1 mg	154 ± 14 μm	29 ± 7	760 ± 30	%76 ± 3
Capsule 8	Fmoc-Pro/2 mg	-	114 ± 14 μm	30 ± 15	-	-
Capsule 9	Fmoc-Pro/2 mg	1 mg	131 ± 15 μm	35 ± 18	780 ± 40	%78 ± 4

a Alginate 2% (w/v).

b CaCl_2_ 3% (w/v).

c 75 μL DMSO.

Quercetin releases from Ca-Alginate (Capsule 5), Ca-Alginate/Fmoc-Y (Capsule 7), and Ca-Alginate/Fmoc-Pro (Capsule 9) microcapsules were carried out in pH = 7.4 PBS solution at 37 °C, 100 rpm stirring rate. In Ca-Alginate microcapsule (Capsule 5, Table 2), drug release from the microcapsules was completed within 7 h. In Ca-Alginate/Fmoc-Y (Capsule 7, Table 2) and Ca-Alginate/Fmoc-Pro (Capsule 9, Table 2) microcapsules, drug release continued up to 24 h. The cumulative release rate of the drug was slower in hybrid Ca-Alginate/Fmoc-Y (Capsule 7, Table 2) and Ca-Alginate/Fmoc-Pro (Capsule 9, Table 2) microcapsules than in Ca-Alginate microcapsules (Capsule 5, Table 2) as expected. The release rate of Ca-Alginate/Fmoc-Pro microcapsule (Capsule 9, Table 2) was slower than Ca-Alginate/Fmoc-Y microcapsule (Capsule 7, Table 2). The drug release rate from microcapsules has been reported to vary depending on the production method, the concentration of solutions used in capsule preparation, and capsule size in various studies.^14–16,19^ In the study by Cadena-Velandia et al., quercetin-loaded Ca-Alginate capsules (0.6 ± 0.1 mm) were produced by the ionic gelation method. When the release of quercetin from Ca-Alginate capsules was examined, it was observed that microcapsules were degraded at pH = 7.4 after 170 min.^14^ When the release of quercetin from microcapsules produced using Alginate/Chitosan at pH = 7.4 was examined, it was found that the drug release was completed after 6 h,^19^ 7 h,^16^ and 24 h.^15^ In our study, Ca-Alginate microcapsule (Capsule 5, Table 2) eroded after 7 h and drug release was completed, whereas in hybrid Ca-Alginate/Fmoc-aa microcapsules (Capsules 7 and 9, Table 2), the release time was extended to 24 h (Figure 9A).

**Figure 9:**
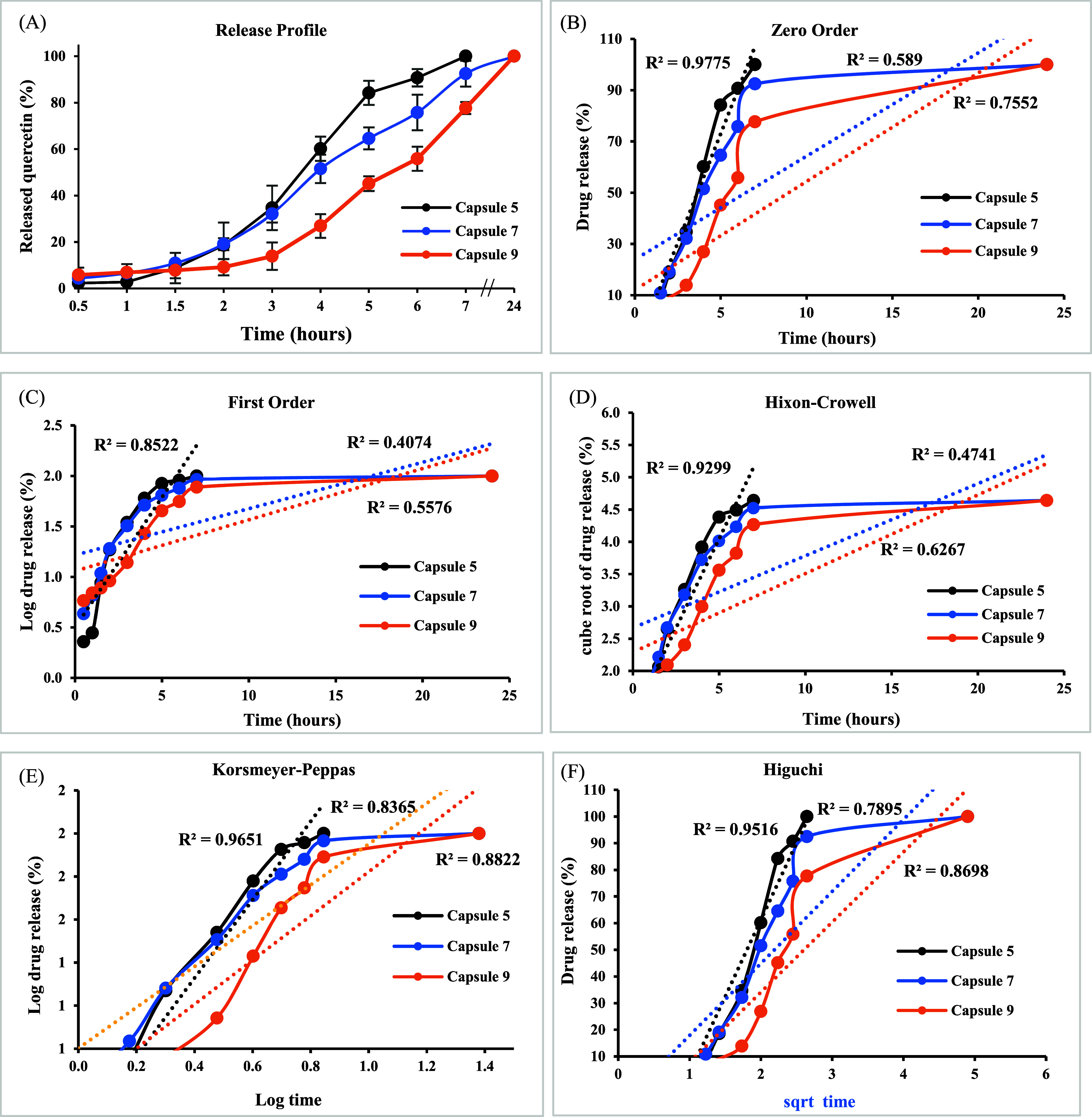
Quercetin release from Ca-Alginate (Capsule 5, Table 2), Ca-Alginate/Fmoc-Y (Capsule 7, Table 2), and Ca-Alginate/Fmoc-Pro microcapsules (Capsule 9, Table 2) (A). Kinetic models of quercetin release: Zero order (B), First order (C), Hixson-Crowell model (D), Korsmeyer–Peppas (E), and Higuchi (F). Each release experiment was performed in triplicate (*n* = 3).

The zero-order, first-order, Hixson-Crowell, Korsmeyer–Peppas, and Higuchi mathematical models^60,61^ were used to determine the release kinetics of quercetin-loaded Ca-Alginate, Ca-Alginate/Fmoc-Pro, and Ca-Alginate/Fmoc-Y microcapsules (Capsules 5, 7, and 9, Table 1) (Figure 9B–F, Table 3). According to the release kinetics models, the models with the highest r^2^ values indicate the mechanism of drug release from microcapsules. In Ca-Alginate microcapsules (Capsule 5, Table 1), the zero-order model was the most appropriate model (Figure 9B). The zero-order release kinetics model illustrates the controlled and sustained release of drugs, especially referring to release conditions such as matrix tablets, transdermal systems, oral osmotic tablets, etc., with low-solubility drugs.^56^ In the Ca-Alginate microcapsules (Capsule 5, Table 1), the highest r^2^ value was found in the Korsmeyer–Peppas model (Figure 9E, Table 3) after the zero-order mathematical model. The value of *n* > 0.89 (Capsule 5, Table 3), obtained according to the Korsmeyer–Peppas model, corresponds to super case II transport, and this value is expressed as relaxed release. It is associated with the dissolution and erosion of the polymer in the buffer solution.^46^ In the Ca-Alginate/Fmoc-Y and Ca-Alginate/Fmoc-Pro microcapsules (Capsules 7 and 9, Table 1), the Korsmeyer–Peppas model was found to be the most appropriate model (Figure 9E, Table 3). The Korsmeyer–Peppas model describes drug release from polymeric systems.^56^ The n (exponent) values in the Korsmeyer–Peppas model for Ca-Alginate/Fmoc-Y and Ca-Alginate/Fmoc-Pro microcapsules (Capsules 7 and 9, Table 3) were in the range of 0.45 < *n* < 0.89, indicating anomalous (non-Fickian) diffusion (capsules 7 and 9, Table 3). This suggests both swelling-controlled and diffusion-controlled abnormal diffusion of the drug release. The studies on alginate microcapsules with similar release kinetics models are available in the literature.^17^ In Ca-Alginate/Fmoc-Y and Ca-Alginate/Fmoc-Pro microcapsules (Capsules 7 and 9, Table 3), the highest r^2^ value after the Korsmeyer–Peppas model was found in the Higuchi model (Figure 9F), indicating release by diffusion.

**Table 3:** Kinetic Models for Quercetin Release from Ca-Alginate, Ca-Alginate/Fmoc-Pro, and Ca-Alginate/Fmoc-Y Microcapsules^a^

	**Zero order**	**First order**	**Hixon-Crowell**	**Korsmeyer–Peppas**		**Higuchi**
**Capsule no**	***r*** ^**2**^	***r*** ^**2**^	***r*** ^**2**^	***r*** ^**2**^ ***n***	***n***	***r*** ^**2**^
5	0.98	0.85	0.93	0.97	1.26	0.95
7	0.59	0.41	0.47	0.84	0.51	0.79
9	0.76	0.56	0.63	0.88	0.65	0.87

a Ca-Alginate, Ca-Alginate/Fmoc-Pro, and Ca-Alginate/Fmoc-Y microcapsules (capsule nos. 5, 7, 9).

According to the release kinetics models, the models expressing sustained and loose release were more dominant in Ca-Alginate microcapsules (Capsule 5, Table 3). In Ca-Alginate/Fmoc-Y and Ca-Alginate/Fmoc-Pro microcapsules (Capsules 7 and 9, Table 3), both swelling and diffusion-controlled release kinetics models were found to be the most appropriate models. It is seen that the release kinetics model changed with the addition of Fmoc-aa to Ca-Alginate microcapsules. In addition, the slower release in hybrid microcapsules (Capsules 7 and 9, Table 3) compared to Ca-Alginate microcapsules (Capsule 5, Table 3) explains the swelling and diffusion-controlled release kinetics. In the study, cumulative release data were analyzed using two-way repeated measures ANOVA, with time as the repeated measures factor and capsule type as the group factor (*p* < 0.05 was considered significant) (Figure 9A).^62^ The analysis results showed that time had a statistically significant and strong effect on cumulative release (*p* < 0.001, η^2^ *_p_* = 0.990). Furthermore, a significant difference was found between capsule types in overall release levels (*p* = 0.035, η^2^ *_p_* = 0.740). A statistically significant interaction between time and capsule type was determined (*p* < 0.001, η^2^ *_p_* = 0.866),^63,64^ indicating that the release profiles of the capsules differed over time.

### 3.5 Swelling Behavior of Microcapsules in Simulated Gastric Fluid and Simulated Colon Fluid

#### 3.5.1 Swelling Behavior in Simulated Gastric Fluid

The swelling behavior of Ca-Alginate (Capsule 4), Ca-Alginate/Fmoc-Y (Capsule 6), Ca-Alginate/Fmoc-Pro (Capsule 8) microcapsules in pH = 1.2 as simulated gastric fluid (HCl solution) was investigated, and light microscopy images were taken at the specified time intervals (0 min, 1 and 7 h) (Figure 10). All of the microcapsules (Capsules 4, 6, and 8) did not deform into spheres for 7 h. However, more shrinkage occurred in hybrid Ca-Alginate/Fmoc-Y (Capsule 6), Ca-Alginate/Fmoc-Pro (Capsule 8) microcapsules than in Ca-Alginate microcapsules (Capsule 4) (Figure 10). In the studies, quercetin-loaded Ca-Alginate microcapsules maintained their spherical form for 7 h in HCl (pH = 1.2) solution.^14^ Carboxylic groups in the structure of alginate are nonionizable (COOH) and insoluble at pH < 3.4.^1^ In our study, all of the microcapsules (Capsules 4, 6, and 8) maintained their spherical form in simulated gastric fluid for up to 7 h. The results demonstrate the durability of drug-loaded microcapsules in the gastric environment during passage through the GIT.

**Figure 10:**
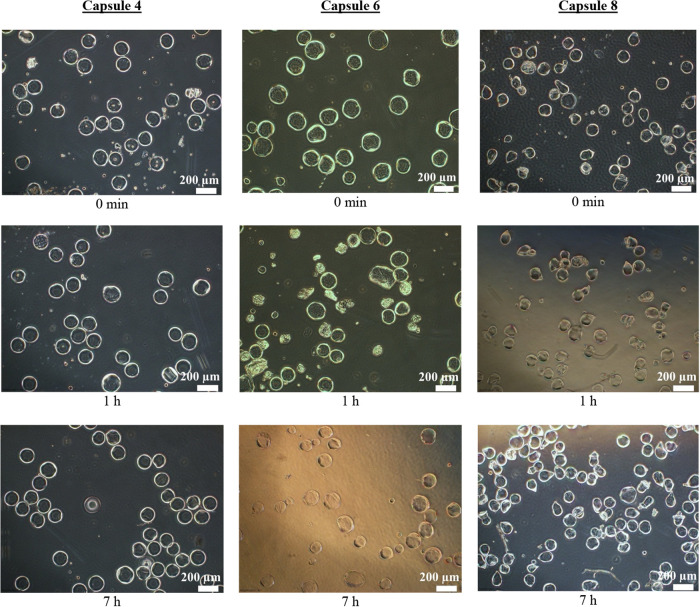
Light microscopy images showing the swelling behavior of Ca-Alginate (Capsule 4), Ca-Alginate/Fmoc-Y (Capsule 6), and Ca-Alginate/Fmoc-Pro (Capsule 8) at pH = 1.2.

#### 3.5.2 Swelling Behavior in Simulated Colon Fluid

The swelling behavior of Ca-Alginate (Capsule 4), Ca-Alginate/Fmoc-Y (Capsule 6), and Ca-Alginate/Fmoc-Pro (Capsule 8) microcapsules was studied at pH 7.4. The lowest percent swelling (80%) in the first hour was found in Ca-Alginate/Fmoc-Y microcapsule (Capsule 6). Ca-Alginate (Capsule 4) and Ca-Alginate/Fmoc-Pro (Capsule 8) microcapsules showed 85 and 90% swelling in the first hour, respectively. When the swelling behavior of the microcapsules was examined by light microscopy, it was observed that in Ca-Alginate microcapsule (Capsule 4), which swelled in the second hour, degradation products were formed (Figure S4). In hybrid microcapsules, microcapsules swelled at the second hour, but no degradation products were observed (Capsules 6 and 8, Figure S4). There were significant differences in the percent swelling of the microcapsules at the third hour. The percent swelling of Ca-Alginate (Capsule 4), Ca-Alginate/Fmoc-Y (Capsule 6), and Ca-Alginate/Fmoc-Pro (Capsule 8) microcapsules at the third hour was 141, 90, and 120%, respectively; the lowest swelling was observed in Ca-Alginate/Fmoc-Y microcapsules (Figure 11). At the sixth hour, microscope images showed that Ca-Alginate microcapsule (Capsule 4) were mostly deformed, lost their spherical form, and showed increased degradation products (Figure S4). Hybrid Ca-Alginate/Fmoc-Pro and Ca-Alginate/Fmoc-Y microcapsules (Capsules 6 and 8), although swelling occurred in the first 6 h, mostly retained their spherical form, and degradation products were considerably less than those of Ca-Alginate microcapsules (Capsule 4). At the 16^th^ hour, the microcapsules (Capsules 4, 6, and 8) were largely deformed (Figure S4). The behavior of the developed microcapsules in simulated colon fluid was evaluated by swelling experiments performed at pH 7.4. The results showed that the hybrid Ca-Alginate/Fmoc-Pro and Ca-Alginate/Fmoc-Y microcapsules (Capsules 6 and 8) retained their spherical form longer and showed lower swelling than Ca-Alginate microcapsule (Capsule 4) (Figures S4 and 11). It was concluded that the incorporation of Fmoc-Pro and Fmoc-Y in microcapsules (Capsules 6 and 8) increased the strength and stability of the microcapsules at pH 7.4, as expected. The carboxyl groups in the alginate ionize at pH > 4.4 (COO^–^), which causes an increase in the electrostatic repulsive force of the negative charges. The resulting increase in repulsive force causes the polymer chain to expand and the hydrophilic matrix to swell, reaching a maximum at pH 7.4.^1^ The slower degradation/erosion of hybrid microcapsules (Capsules 6 and 8) at pH 7.4 compared to Ca-alginate microcapsule (Capsule 4) supports the cross-linking of carboxyl groups in alginate and Fmoc-aa with Ca^2+^ ions.

**Figure 11:**
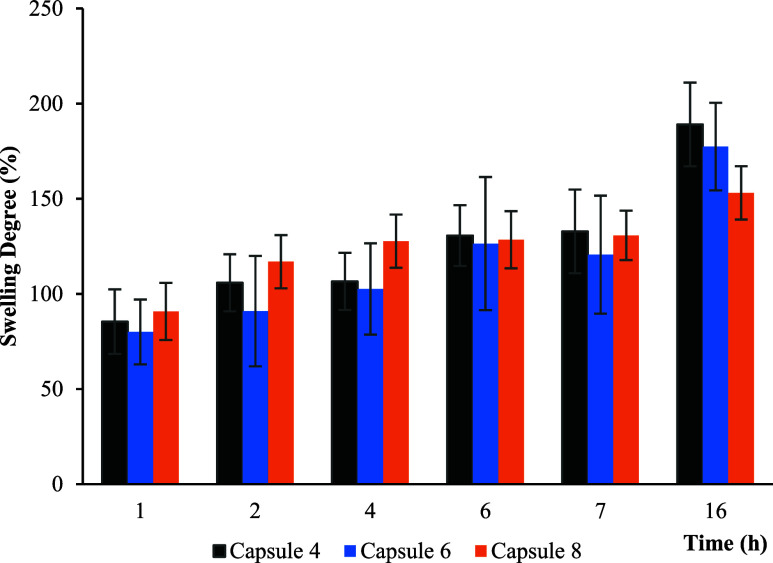
Swelling degree of Ca-Alginate (Capsule 4), Ca-Alginate/Fmoc-Y (Capsule 6), and Ca-alginate/Fmoc-Pro (Capsule 8) microcapsules.

*In vitro* swelling at pH 1.2 and swelling and release studies conducted at 7.4 provide simplified models of the stomach and colon environments, respectively. However, these media do not contain digestive enzymes, bile salts, or intestinal microbiota and, therefore, cannot fully replicate the complexity of *in vivo* conditions.^65^ Despite these limitations, the stability of the microcapsules observed at pH 1.2 suggests effective protection of the encapsulated drug in the gastric environment, while the release profile at pH 7.4 supports drug release in the target region, the colon. Accordingly, these *in vitro* release and swelling studies were performed to provide an initial physicochemical evaluation and to estimate the potential applicability of the system for colon-targeted drug delivery.^19,66^

## 4 Conclusion

In this study, quercetin-loaded Ca-Alginate, Ca-Alginate/Fmoc-Y, and Ca-Alginate/Fmoc-Pro hybrid microcapsules were successfully produced using the electrospray method. Unlike previous studies in the literature, Fmoc-Y and Fmoc-Pro were integrated into the alginate structure for the first time and prepared in the form of microcapsules, and their quercetin release behavior was studied in detail. When evaluating the quercetin release profiles, it was observed that release was completed within approximately 7 h in Ca-Alginate microcapsules, whereas the release time was extended up to 24 h in Ca-Alginate/Fmoc-Y and Ca-Alginate/Fmoc-Pro hybrid microcapsules. EE values were determined as 90 ± 10, 76 ± 3, and 78 ± 4% for Ca-Alginate, Ca-Alginate/Fmoc-Y, and Ca-Alginate/Fmoc-Pro microcapsules, respectively. Although sustained and controlled release profiles were successfully achieved in the hybrid microcapsules, the lower EE values observed relative to pure Ca-Alginate microcapsules may be attributed to the incorporation of Fmoc-Y and Fmoc-Pro, which likely altered the physicochemical properties of the precursor solution during the electrospray process, thereby affecting encapsulation performance. When release kinetics models were examined, it was determined that the most suitable mathematical model for Ca-Alginate microcapsules is the zero-order model, while the Korsmeyer–Peppas model is the most suitable for Ca-Alginate/Fmoc-Y and Ca-Alginate/Fmoc-Pro hybrid microcapsules. This result indicates that the release mechanism in hybrid systems depends not only on diffusion but also on complex interactions within the matrix structure. When the swelling behavior of the microcapsules under pH = 1.2 (SGF condition) was examined using light microscopy, all microcapsules were observed to shrink. In contrast, in the pH = 7.4 (SCF) environment, Ca-Alginate microcapsules underwent significant deformation within 6 h, while Ca-Alginate/Fmoc-Y and Ca-Alginate/Fmoc-Pro microcapsules largely retained their spherical shape during the same period. Although deformation was observed in all microcapsules after 16 hours, the hybrid microcapsules exhibited more limited structural degradation. These findings demonstrate that the addition of Fmoc-aa increases the structural strength of the microcapsules. Furthermore, rheological analyses demonstrated that the incorporation of Fmoc-Y increased the viscosity and mechanical strength of the system, while simultaneously rendering the network more prone to deformation. In contrast, the addition of Fmoc-Pro resulted in a more balanced rheological profile and promoted the formation of a structurally more stable network. SEM analyses support these results, showing that Ca-Alginate/Fmoc-Y and Ca-Alginate/Fmoc-Pro microcapsules exhibit a more compact, homogeneous morphology with a narrower pore structure. These morphological differences observed in hybrid systems compared to pure Ca-Alginate microcapsules explain the more effective retention of quercetin within the matrix and the prolongation of its release. Quercetin has significant therapeutic potential in the treatment of colon-related disorders such as inflammatory bowel disease (IBD). The microcapsule system developed in this study contributes to increasing treatment efficacy by slowing down and prolonging the release of quercetin. Furthermore, Ca-Alginate/Fmoc-Y and Ca-Alginate/Fmoc-Pro hybrid microcapsules can be considered as promising carrier systems for the controlled release of drugs with low water solubility, such as quercetin.

## Supplementary Material



## Notes

SEM analysis of lyophilized empty and quercetin-loaded Ca-Alginate (Capsules 4 and 5), Ca-Alginate/Fmoc-Y (Capsules 6 and 7), Ca-Alginate/Fmoc-Pro microcapsules (Capsules 8 and 9), quercetin calibration curve, and the component of SEM device for samples taken under wet conditions (PDF)
