# Supplementary material for: Electrosprayed Alginate–Fmoc Amino Acid Microcapsules for Quercetin Loading and Release
Source: ACS Omega. 2026 Jun 15;11(25):36813–27. doi: 10.1021/acsomega.6c00004 (PMC13325145; doi:10.1021/acsomega.6c00004)
Supplement: Supplementary file 1 [file ao6c00004_si_001.pdf]

SUPPORTING INFORMATION

ELECTROSPRAYED ALGINATE–FMOC AMINO ACID MICROCAPSULES FOR QUERCETIN LOADING AND RELEASE

Hatice Asri[1], *Serap Mert*[2-4]*

[1] Department of Biomedical Engineering, Faculty of Technology, Kocaeli University, 41001, Kocaeli, Türkiye

[2] Department of Chemistry, Faculty of Arts and Sciences, Kocaeli University, 41001, Kocaeli, Türkiye

[3] Department of Polymer Sci. and Technol., Kocaeli University, 41001, Kocaeli, Türkiye

[4] Center for Stem Cell and Gene Therapies Res. and Pract., Kocaeli University, 41001, Kocaeli, Türkiye

*E-mail: serap.mert@kocaeli.edu.tr.

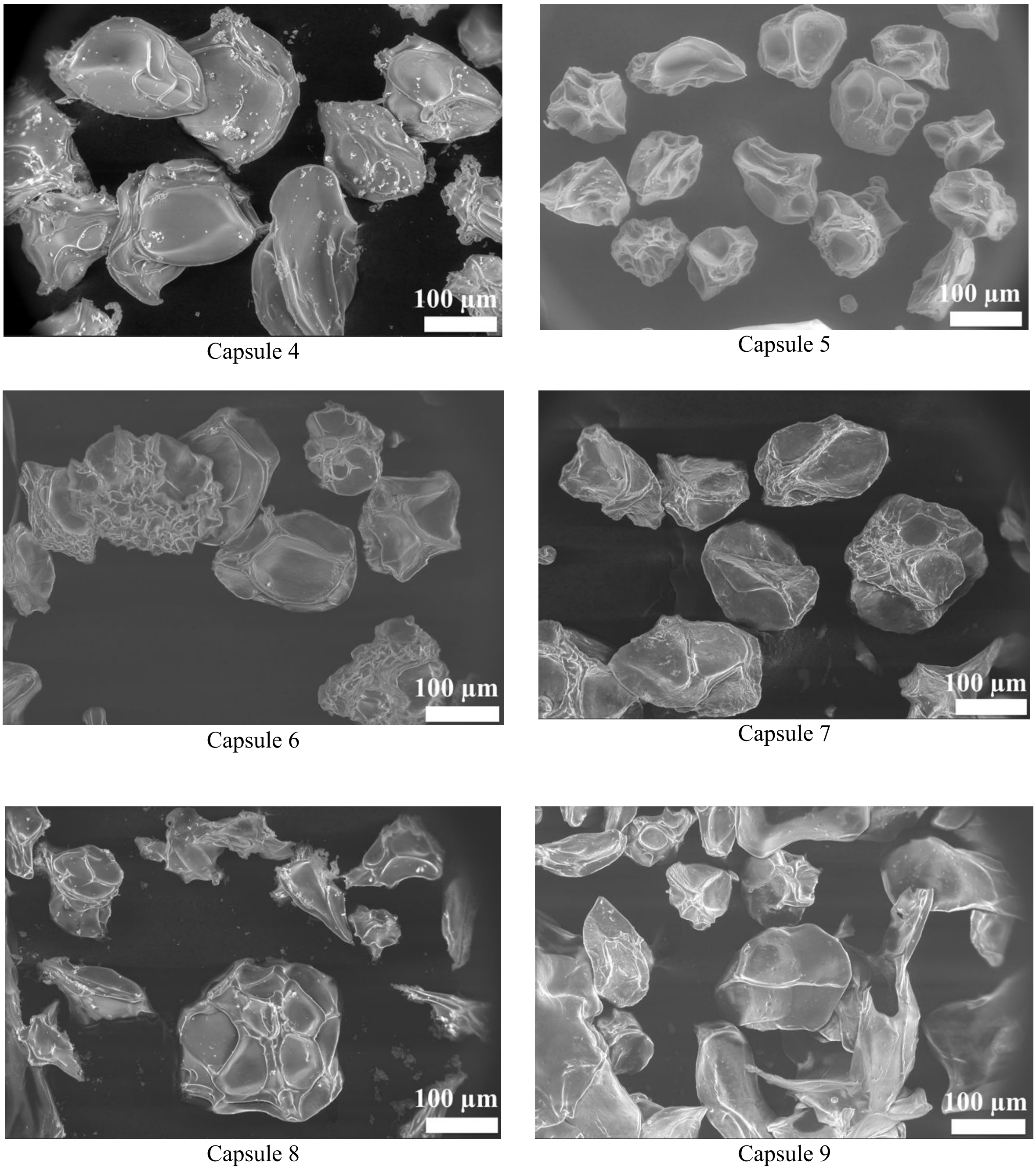

FIGURE S1. SEM Analysis of lyophilized empty and quercetin loaded Ca-Alginate (Capsule 4 and 5), Ca-Alginate/ Fmoc-Y (Capsule 6 and 7), Ca-Alginate/Fmoc-Pro (Capsules 8 and 9) microcapsules

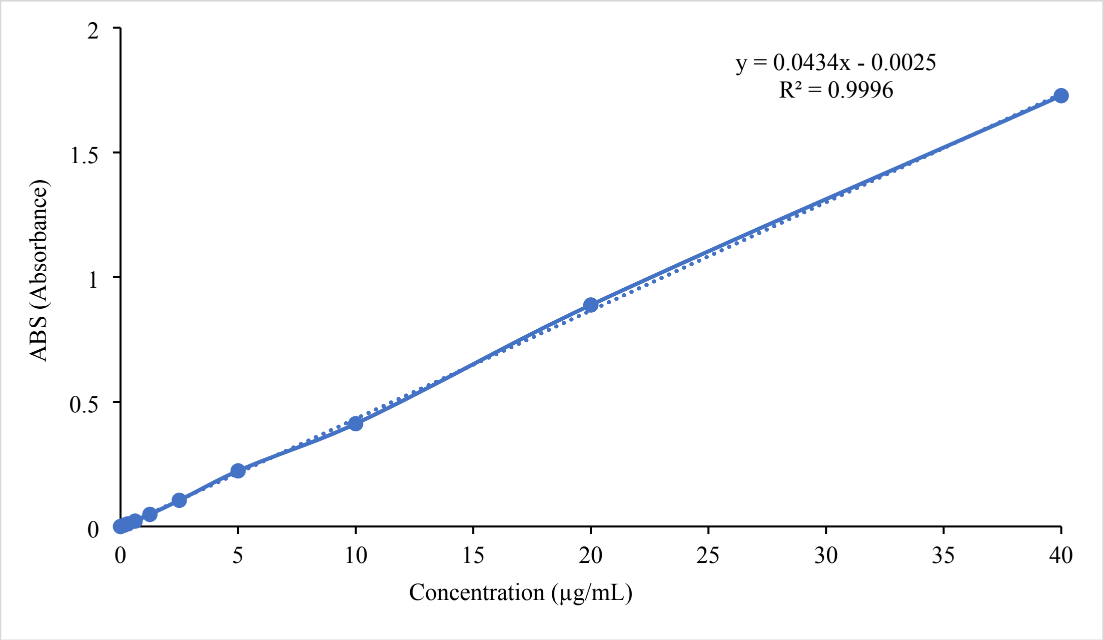

FIGURE S2. Quercetin Calibration Curve

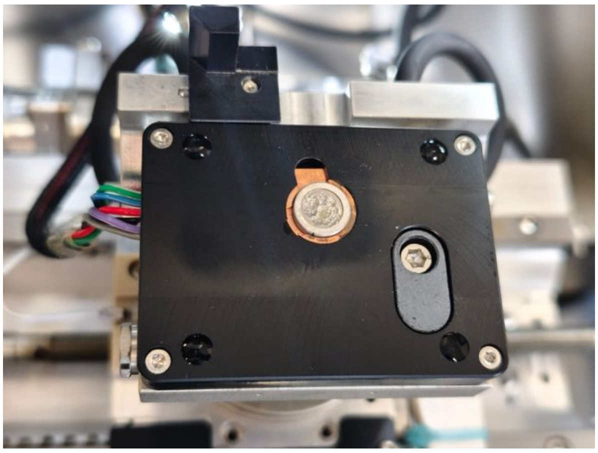

FIGURE S3. The component of SEM device for samples taken under wet conditions

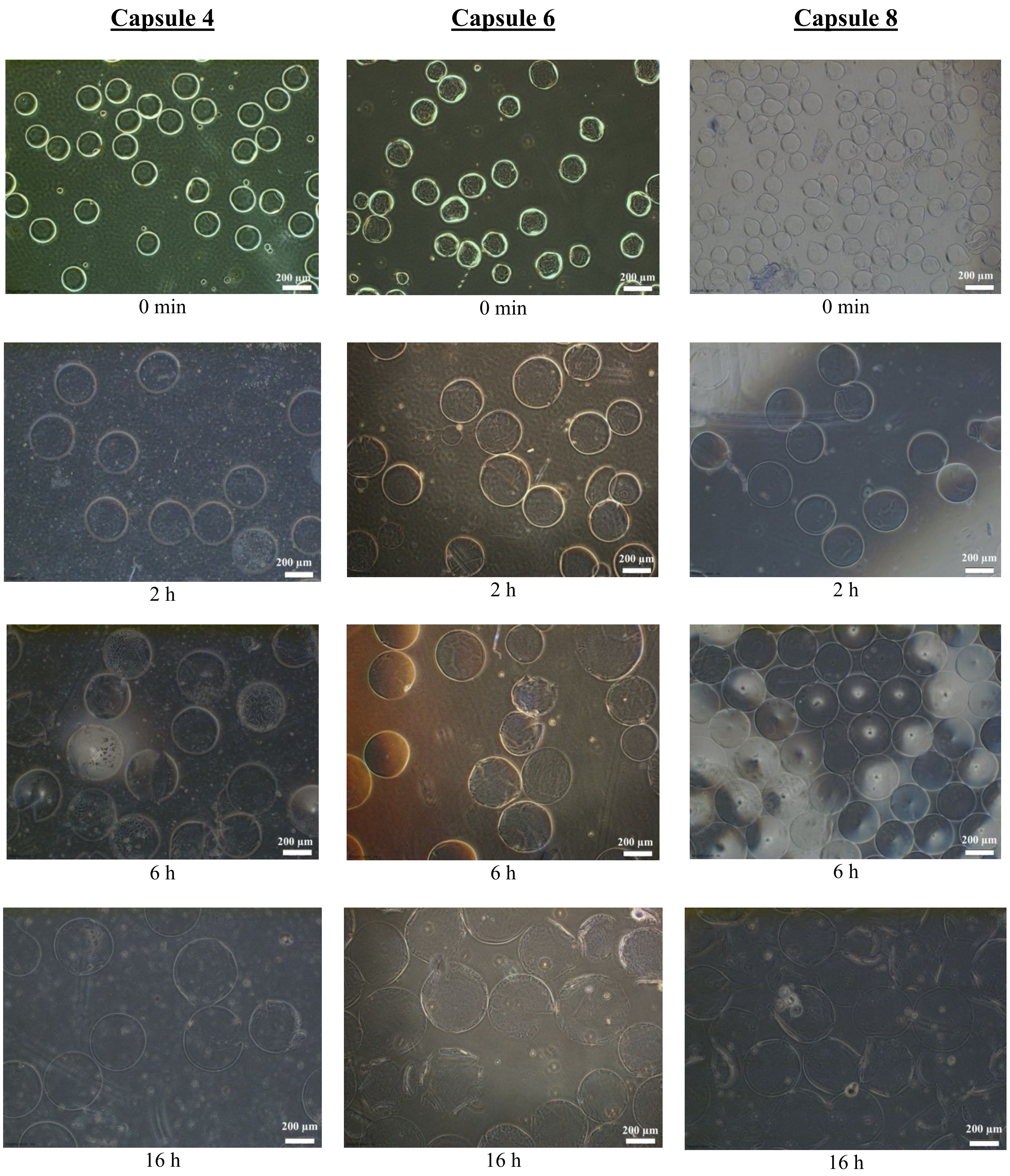

Figure S4. Microscope images of the swelling behavior of Ca-Alginate (Capsule 4), Ca-Alginate/Fmoc-Y (Capsule 6), Ca-Alginate/ Fmoc-Pro (Capsule 8) microcapsules at pH=7.4 at different times
